# Effect of pre-pubertal growth rate of Sohagi ram lambs on some physiological parameters and sexual behavioral patterns at puberty

**DOI:** 10.1590/1984-3143-AR2021-0104

**Published:** 2021-12-01

**Authors:** Mohamed Youssef Elaref, Gamal Mahmoud Solouma, Doaa Ahmed Abdel-latif

**Affiliations:** 1 Department of Animal Production, Faculty of Agriculture, Sohag University, Sohag, Egypt

**Keywords:** pubertal age and weight, testosterone concentration, testes measurements, pubertal behavior activities

## Abstract

Thirty healthy Sohagi ram lambs with an average age of 188.6±7.3 days were used to study the effect of pre-pubertal growth rate on some physiological parameters and sexual behavioral patterns at puberty. Ram lambs were divided into three groups (10 animals per each group) according to the previous growth rate until 6 months of age. Groups were marked as fast, medium and slow growing. Animal groups were housed in closed barns with access to an open area. Results showed that age and weight of ram lambs at puberty were significantly affected (P<0.05) by the pre-pubertal growth rate. Ram lambs in the fast growing group were reached to onset puberty firstly at 272.6 days with body weight (BW) 37.1 kg on average then ram lambs in medium group (284.8 days with BW 32.7 kg), while ram lambs in slow growing group were the last (314.1 days with BW 32.5 kg). Blood‎ testosterone‎ concentration at puberty was not significantly different among growing groups (1.494± 0.03 ng/ml on average, ranged from 1.287 to 1.902 ng/ml). Testes measurements from 6 months of age until puberty show that ram lambs in fast growing group had the highest values of testes length, circumference and volume followed by those in medium and slow growing group. Sexual behavioral observation showed that flehmen and mounting behavior were significantly higher for ram lambs in fast growing group (5.63 and 6.75 number/12h) than slow growing group (4.25 and 5.38 number/12h) while in medium growing group were intermediate (4.88 and 5.88 number/12h). From these findings, could be concluded that age, weight and sexual behavioral patterns of Sohagi ram lambs at puberty were affected by pre-pubertal growth rate, and the breeders should strive to achieve good growth rates for their lambs before puberty which led to improving reproductive performance.

## Introduction

The efficiency of sheep production particularly meat production in developing countries is important and depends mainly on the reproductive efficiency of the sheep. Male lambs with early puberty and distinctive sexual behavior can improve flock fertility during breeding and indirectly genetic improvement (Ibarra et al., 2000). Moreover, characterization of puberty and early sexual development is a valuable tool for genetic selection within the breed (El-Shahat et al., 2014). Researchers interested in age at puberty are agreed that achieving an early age at puberty is associated with the time of birth and the nutritional planes (Khalifa et al., 2013). Moulla et al. (2018) revealed that ‎the complexity to define ‎accurately onset of puberty particularly in species with seasonal ‎reproduction activity. The interaction between body weight, testis ‎growth and testosterone secretion during the pre-pubertal stage is the key factor influencing puberty (Martinez et al., 2012). Sohagi sheep is one of the most prevalent types of sheep in Upper Egypt which is exploited by small farmers to improve their livelihoods and annual income (Galal et al., 2002). Birth weight and growth performance of Sohagi ram lambs pre and post-weaning were significantly affected by environmental factors (such as birth type, ewe parity and lambing season) that play an important role in expressing the inherent potential of Sohagi lambs (Elaref et al., 2020). Othman et al. (2016) suggested that the situation of the Sohagi sheep has to be carefully considered to prevent possible genetic dilution of the breed because Sohagi sheep are of crucial importance for the livelihoods of farmers living in the poorest areas of the country. They should be given priority for the conservation and establishment of breed schemes. Full information on reproductive performance of Sohagi ram lambs and their sexual behavior development are lacking. This work aimed to study the effect of pre-pubertal growth rate on some physiological parameters and sexual behavioral patterns of Sohagi ram lambs at puberty.

## Materials and methods

### Ethical statement

The current research work was carried out in accordance with the guidelines of the Institutional Animal Care and Use Committee (IACUC) of the Faculty of Veterinary Medicine, Cairo University, Egypt (VetCU05192019041).

### Animal and management

The present study was conducted at experimental sheep farm of animal production department, Faculty of Agriculture, Sohag University, Egypt from December 2018 to April 2019. Sohag is one of the rural governorates in Upper Egypt (latitude 26.36°, longitude 31.38° and elevation above sea level 68.70 m), characterized by a dry desert climate (Zohry and Ouda, 2016). Sohagi sheep breed is characterized by breeding throughout the year. Healthy thirty single births of Sohagi ram lambs with an average age of 188.6±7.3 days were divided into three groups (10 animals per each group) according to the previous growth rate from birth until 6 months of age. Groups were marked as fast, medium and slow growing. Animal groups were housed in closed barns with access to an open area and fed according to National Research Council‎ (2007) recommendations depending on their growth stage. Freshwater was available all the time of the day from a fixed drinking trough.

### Blood samples and laboratory analysis

Blood samples were obtained from lambs via jugular vein puncture at 10:00 AM once biweekly until the end of the experiment (11 months of age). Plasma was separated by centrifugation at 3000 rpm for 15 min within an hour of collection, after that the obtained plasma was transferred into a clean Eppendorf tube and stored until hormone analysis at -20ºC to estimate the concentration of plasma testosterone hormone. Testosterone hormone was analyzed using radioimmunoassay kits (Diagnostic Product Company, LOS Angeles, CA).

### Pubertal behavior observation

The experimental groups were video recorded for 12h/day (daylight) using a digital Surveillance video recording system (Digital video recorder 8 channel, three outdoor cameras, three indoor cameras and digital display monitor) for three continuous months, and one day weekly was chosen randomly during the observation period to investigate the sexual behavioral patterns of the ram lambs in the three growing groups. According to Clemente et al. (2013) and Kridli et al. (2007) behavioral events used to quantify ram lambs sexual behavior were included: a) Sniffing; b) Flehmen c) Foreleg kicking d) mount attempts; e) number of mounts with or without penile extension.

### Testes morphometric measurements of ram lambs

All testes morphometric measurements of ram lambs were determined once biweekly until the end of the experiment. Scrotal circumference was measured by using a flexible tape around the widest point of the ‎testes (maximum circumference of the paired testes). The measurement ‎of testis length was taken at the point of top and bottom dimensions of ‎testis with calipers. Testes volume (cm^3^) was calculated according to the ‎equation given by El-Zelaky et al. (2011):‎


$$\text{Testes volume}\left(cm^{3}\right)=0.0396\times \text{average testis length}\times {\left(\text{scrotal circumference}\right)}^{2}$$ (1)

### Statistical analysis

Data were analyzed using the PROC MIXED for repeated measurements of SAS (SAS, 9.3) and the results presented as Least Squares Means (LSM). The statistical model included the fixed effects of the lambs group (fast, medium and slow growing), and sample time (biweekly sample from the beginning until the end of the experiment). Differences between LSM were determined with the PDIFF option of SAS. Statistical model used for analyze all obtained data was: Y*_ijk_* = µ + G*_i_* + T*_j_* + ε*_ijk_* Where Y*_ijk_* is the dependent variable (Age and weight at puberty, testosterone level, testes morphometric measurements and pubertal sexual behavior of ram lambs), µ is the overall mean, G*_i_* is the fixed effect of ram lambs group, T*_j_* the fixed effect of sample time and ε *_ijk_* is the random residual error.

## Results

The effect of pre-pubertal growth rate of ram lambs on age and weight at puberty of Sohagi ram lambs was shown in Table 1. The upper part of the table displays growth performance during the first 6 months of age for the three groups of ram lambs (fast, medium and slow growing). Birth weight did not differ significantly (P<0.05) in experimental groups while average daily gain (g) and total weight gain (kg) during the first 6 month of age were significant different (P<0.05), the fast growing group had the highest average daily gain and total weight gain (168.8 g and 30.4 kg) than the medium growing group (140.5 g and 25.3 kg) while the slow growing group had the lowest average daily gain and total weight gain (119.3 g and 21.5 kg).

**Table 1:** Effect of pre-pubertal growth rate (fast, medium and slow growing) on age, weight, testosterone concentration and testes measurements of Sohagi ram lambs at puberty.

	**Ram lambs groups**			**SEM**	***P* value**
	**Fast** **growing**	**Medium** **growing**	**Slow** **growing**		
Birth weight (kg)	3.11	3.12	3.04	0.02	0.618
Weight at 6 month (kg)	33.5^a^	28.4^b^	24.5^c^	0.75	0.001
Total weight gain (kg)	30.4^a^	25.3^b^	21.5^c^	0.75	0.001
Average daily gain (g)	168.8^a^	140.5^b^	119.3^c^	4.17	0.001
Pubertal characteristics					
Age at puberty (day)	272.6^a^	284.8^a^	314.1^b^	3.47	0.001
Weight at puberty (kg)	37.1^a^	32.7^b^	32.5^b^	0.50	0.001
Testosterone at puberty (ng/ml)	1.522	1.492	1.468	0.03	0.813
Testes measurements at puberty					
− Test length (cm)	13.25	13.10	12.80	0.12	0.280
− Test circumference (cm)	24.55	24.30	24.05	0.10	0.158
− Test volume (cm^3^)	316.74	306.64	293.47	4.44	0.119

SEM = Standard error of mean. ^abc^Mean values with a different superscript in the same row indicate significant difference (P<0.05).

Age and weight of ram lambs at puberty were significantly affected (P<0.05) by pre-pubertal growth rate of ram lambs. Ram lambs in fast growing group were reached to onset puberty firstly (272.6 days) then ram lambs in medium group (284.8 days), while ram lambs in slow growing group were the last (314.1 days). Also, ram lambs in fast growing group were the heaviest in weight (P<0.05) at puberty than ram lambs in medium and slow growing group (37.1 vs. 32.7 and 32.5 kg, respectively) (Table 1).

**Figure 1:**
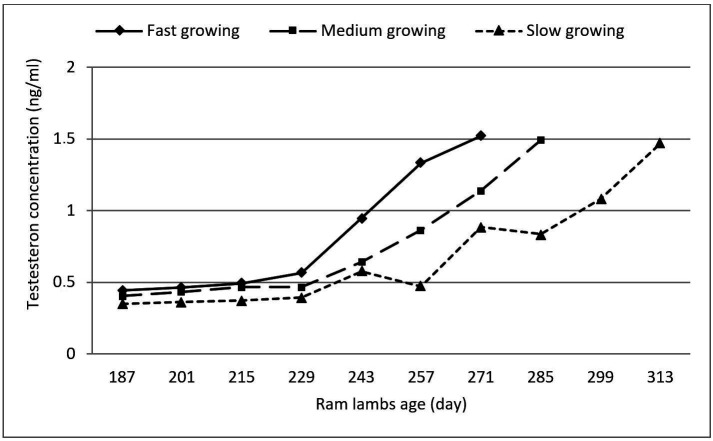
Blood testosterone‎ concentration ‎of Sohagi ram lambs groups (fast, medium and slow growing) from 6 month of age until puberty.

Blood testosterone concentration of Sohagi ram lambs in growing groups was not significantly different at puberty (1.494± 0.03 ng/ml on average, ranged from 1.287 to 1.902 ng/ml) (Table 1 and Figure 1). Blood testosterone concentration before puberty of ram lambs had linear increase trend in the values with age and didn’t exceed 1.0 ng/ml then a noticeable rapid and stable increase in testosterone concentration (over 1.0 ng/ml) occurs close to sexual puberty time (Figure 1).

**Figure 2:**
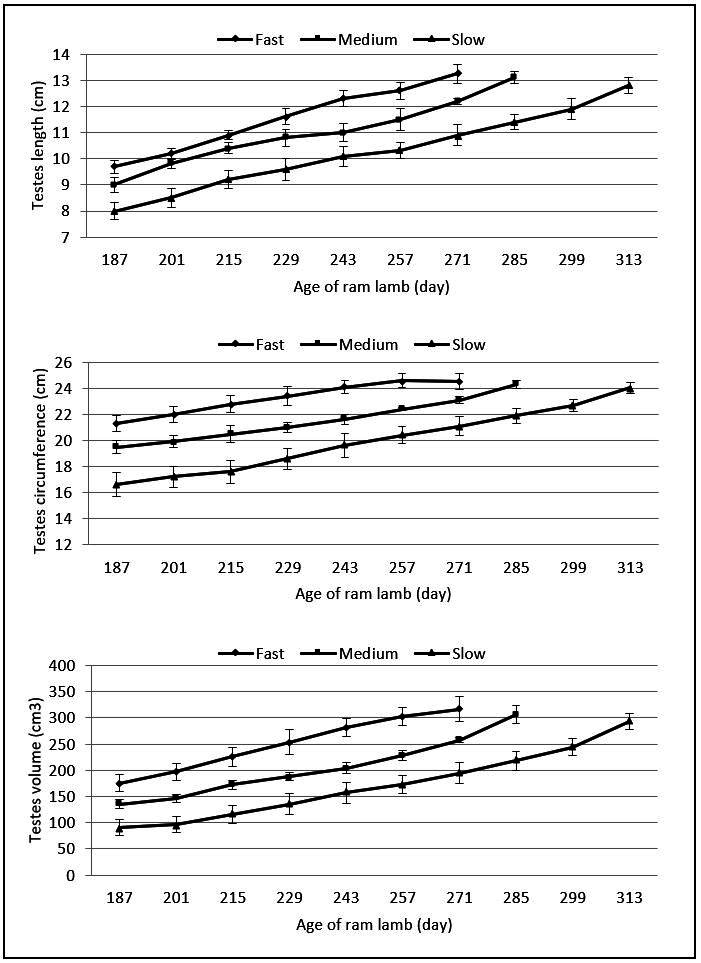
Testes measurements ‎of Sohagi ram lambs groups (fast, medium and slow growing) from 6 month of age until puberty.

Testes measurements ‎of Sohagi ram lambs groups (fast, medium and slow growing) from 6 months of age until puberty were shown in Table 1 and Figure 2. Biweekly measurements of testes length (cm), circumference (cm) and volume (cm^3^) of ram lambs in growing groups were significantly different (P<0.05). The ram lambs in fast growing group had the highest values of testes length, circumference and volume followed by ram lambs in medium growing group and ram lambs in slow growing group had the lowest values. On the other hand, testes measurements of ram lambs at puberty were not significantly different (P<0.05) in growing groups.

The effect of pre-pubertal growth rate (fast, medium and slow growing) on pubertal sexual behavior of Sohagi ram lambs was displayed in Table 2. All studied pubertal sexual behavior patterns were significantly affected (P<0.05) by pre-pubertal growth rate of ram lambs. Frequencies of sniffing and foreleg kicking behavior of ram lambs in fast growing group were significantly (P<0.05) higher (8.63 and 9.88 number/12h) than in medium (6.88 and 7.63 number/12h) and slow growing groups (5.75 and 7.25 number/12h). Flehmen and mounting behavior were significantly higher for ram lambs in fast growing group (5.63 and 6.75 number/12h) than slow growing group (4.25 and 5.38 number/12h) while in medium growing group were intermediate (4.88 and 5.88 number/12h). On the other hand, numbers of mount attempts were significantly lower for ram lambs in fast growing group than in medium and slow growing groups (2.75, 3.38 and 3.63 number/12h, respectively).

**Table 2:** Effect of pre-pubertal growth rate (fast, medium and slow growing) on pubertal sexual behavior of Sohagi ram lambs.

**Sexual behaviour aspects** **(number/12h)**	**Ram lambs groups**			**SEM**	***P* value**
	**Fast** **growing**	**Medium** **growing**	**Slow** **growing**		
Sniffing	8.63^a^	6.88^b^	5.75^b^	0.40	0.006
Flehmen	5.63^a^	4.88^ab^	4.25^b^	0.23	0.046
Foreleg kicking	9.88^a^	7.63^b^	7.25^b^	0.46	0.036
Mount attempts	2.75^b^	3.38^a^	3.63^a^	0.12	0.006
Mounting	6.75^a^	5.88^ab^	5.38^b^	0.23	0.033

SEM = Standard error of mean. ^abc^Mean values with a different superscript in the same row indicate significant difference (P<0.05).

## Discussion

Sohagi sheep are well coped with the hot and dry conditions in Upper Egypt; however, this native breed remains not well studied and little previous studies are available, especially concerning the reproduction physiology. The knowledge and mastery of reproduction particularly in males are one of the cornerstones in breeding and management programs. Elhammali et al. (2013) reported that the differences observed in terms of onset puberty are attributed to several factors (i.e. breed, climate and nutrition management). The effect of pre-pubertal growth rate of Sohagi ram lambs on some physiological parameters and sexual behavioral patterns at puberty were studied.

The obtained results show that Sohagi ram lambs in fast growing group were reached the onset puberty firstly with heaviest weight than those in medium and slow growing groups. The same result was found by Zarkawi and Al-Daker (2016) who conducted a similar study on Awassi ram lambs to characterize body weight and some reproductive parameters at puberty in fast and weak growing ram lambs. They found that fast growing ram lambs reached firstly to puberty with high body weight than weak growing ram lambs. In the same regard, numerous researchers ‎determined age and weight at puberty of Egyptian sheep breeds and some of their crosses. Eissa et al. (2013) reported that male lambs of crossbred breed (½ Awassi × ½ Barki) reached to puberty at shorter age and heavier weights than Barki breed. Furthermore, Kridli et al. (2006)‎‏ study the age and weight of Awassi ram lambs and their crosses with Charollais and Romanov. He found that the two crosses with Awassi were reached to puberty faster than Awassi breed while weight ‎at puberty of the three genotypes was not significantly. This indicates that the occurrence of puberty in Sohagi ram lambs is strongly related to their pre-pubertal growth rates.

Generally, the present results show that the age of sexual puberty in Sohagi ram lambs is relatively late (290.5 days on average) than some other Egyptian sheep breeds and the reason for this must be studied.

Blood testosterone concentration of Sohagi ram lambs in growing groups was not significantly different at puberty. In contrast, Mukasa-Mugerwa and Ezaz (1992) and Yılmaz (2006) observed that testosterone concentrations in ram lambs at puberty were affected by body weight and age. On the other hand, blood‎ testosterone concentration of Sohagi ram lambs before puberty had linear increase trend in the values with age and didn’t exceed 1.0 ng/ml. These results were compatible with those obtained by Zarkawi and Al-Daker (2016) and Khalifa et al. (2013). Also, Maksimović et al. (2016) established a linear increase in testosterone level of male lambs in the ‎period from 3 to 7 months of age.

The results of testes measurements ‎of Sohagi ram lambs were agreement with those by Maksimović et al. (2016); Andrade et al. (2018); Khalifa et al. (2013) and Koyuncu et al. (2011) who found a positive correlation between pre-pubertal lamb testosterone hormone levels and subsequent either testes size or age, meaning that the male lambs with bigger testes ‎produced more testosterone levels than the lambs with smaller testes. In addition, Elmaz et al. (2007) found that testosterone concentration was 0.4 and 2.5 ‎ng/ml when testes volume 17.3 and 211.9 cm^3^ with 100 and 200 days ‎of age, respectively. So, it could be concluded that testosterone concentration was affected by body weight and age of lambs and increased testosterone concentration in ram lambs might be attributed to the rising volume of testes.

The results of the current study showed that pubertal sexual behavior patterns were improved significantly with increasing pre-pubertal growth rate of ram lambs. This result was supported by the results of Simitzis et al., (2006) who reported that age and weight of rams had a significant effect on its sexual behavior like sniffing and nudging and concentration of testosterone hormone may be a contributing factor. Similar findings were obtained by Mohamed et al. (2016) who found that using rice straw treated with enzymes or effective microorganisms as energy sources in Ossimi ram lambs dietary were enhanced growth rate, testosterone level and sexual activity of ram lambs pre and at puberty. Hafez and Hafez (2013) and Mostafa and Farghal (2019) reported that testosterone hormone has a fundamental role in the reproductive behavior of rams and their secondary sexual characteristics.

## Conclusion

In conclusion, results from the present study revealed that physiological parameters and sexual behavioral patterns of Sohagi ram lambs at puberty were affected by pre-pubertal growth rate, and the breeders should strive to achieve good growth rates for their lambs before puberty which will certainly lead to an improvement in reproductive performance..

So, it could be recommended that the growth rate of lambs before puberty is one of the most important and influential factors affecting the development of puberty in sheep. This needs the attention of breeders to achieve the best characteristics of puberty at a younger age with an adequate weight of the breed and consequently improve the production of sheep.
